# Genotype-dependent *N*-glycosylation and newly exposed *O*-glycosylation affect plasmin-induced cleavage of histidine-rich glycoprotein (HRG)

**DOI:** 10.1016/j.jbc.2024.105683

**Published:** 2024-01-24

**Authors:** Yang Zou, Matti F. Pronker, J. Mirjam A. Damen, Albert J.R. Heck, Karli R. Reiding

**Affiliations:** 1 Biomolecular Mass Spectrometry and Proteomics, Bijvoet Center for Biomolecular Research and Utrecht Institute for Pharmaceutical Sciences, Utrecht University, Utrecht, The Netherlands; 2 Netherlands Proteomics Center, Utrecht, The Netherlands

**Keywords:** *N*-glycosylation, *O*-glycosylation, glycoproteomics, histidine-rich glycoprotein, plasmin-induced cleavage, human plasma, CHO cells, HEK293 cells

## Abstract

Histidine-rich glycoprotein (HRG) is an abundant plasma protein harboring at least three *N*-glycosylation sites. HRG integrates many biological processes, such as coagulation, antiangiogenic activity, and pathogen clearance. Importantly, HRG is known to exhibit five genetic variants with minor allele frequencies of more than 10%. Among them, Pro204Ser can induce a fourth *N*-glycosylation site (Asn202). Considerable efforts have been made to reveal the biological function of HRG, whereas data on HRG glycosylation are scarcer. To close this knowledge gap, we used C18-based LC–MS/MS to study the glycosylation characteristics of six HRG samples from different sources. We used endogenous HRG purified from human plasma and compared its glycosylation to that of the recombinant HRG produced in Chinese hamster ovary cells or human embryonic kidney 293 cells, targeting distinct genotypic isoforms. In endogenous plasma HRG, every *N*-glycosylation site was occupied predominantly with a sialylated diantennary complex–type glycan. In contrast, in the recombinant HRGs, all glycans showed different antennarities, sialylation, and core fucosylation, as well as the presence of oligomannose glycans, LacdiNAcs, and antennary fucosylation. Furthermore, we observed two previously unreported *O*-glycosylation sites in HRG on residues Thr273 and Thr274. These sites together showed more than 90% glycan occupancy in all HRG samples studied. To investigate the potential relevance of HRG glycosylation, we assessed the plasmin-induced cleavage of HRG under various conditions. These analyses revealed that the sialylation of the *N*- and *O*-glycans as well as the genotype-dependent *N*-glycosylation significantly influenced the kinetics and specificity of plasmin-induced cleavage of HRG.

Histidine-rich glycoprotein (HRG), also known as histidine–proline-rich glycoprotein, is a plasma glycoprotein that is mainly produced in the liver. The protein is abundant in plasma with a concentration of 100 to 150 μg/ml (1), and it has been reported to bind various ligands, such as coagulation factor XIIa (2), plasminogen (3), immunoglobulin G (4), phospholipids (5), C1q (4), heparin (6), heme (7), fibrinogen (8), and Zn^2+^ ions (9). Because of this broad binding profile, HRG has also been associated with the regulation of many biological processes, including pathogen clearance (5, 10), cell adhesion (11), angiogenesis (12, 13, 14), coagulation (11, 15), and fibrinolysis (11). Plasmin has the ability to cleave human HRG *in vitro* and *in vivo*, a process that is crucial for the interaction of HRG with cell surface heparan sulfate, as well as its binding to necrotic cells and plasminogen, which plays an important role in regulating HRG (16). A total of five gene variant mutations of HRG have been reported with minor allele frequencies (MAFs) of more than 10%, namely Ile180Thr (MAF = 13.52%), Pro204Ser (MAF = 49.14%), His340Arg (MAF = 20.49%), Arg448Cys (MAF = 27.32%), and Asn493Ile (MAF = 65.06%) worldwide (17, 18), meaning that these HRG variants exist, either as homozygote or heterozygote, within a substantial portion of the human population. In our previous study, based on serum from 44 individuals and approximately 2500 genomes from the 1000 Genomes Project, we found that these five gene variant mutations do not occur independently. For instance, several mutations at different sites are fully mutually exclusive, exemplified by the relationship between Cys448 and Asn493, whereas others are highly correlated, such as Pro204 and Asn493 (19).

Glycosylation is an important post-translational protein modification and is associated with biological processes, such as membrane receptor signaling, protecting from immune recognition, and triggering of endocytosis and phagocytosis (https://academic.oup.com/glycob/article/27/1/3/2527575). HRG typically contains three *N*-glycosylation sites on residues Asn63, Asn125, and Asn344, all supposedly having several functional effects. For instance, Asn63 and Asn125 are situated in the N-terminal domain of HRG, which interacts with heparan sulfate and plays an important role in regulating cell proliferation (20, 21). In addition, the role of HRG in hepatocellular cancer appears dependent on its *N*-glycosylation status, especially that of Asn125, which may play a key competitive role in the interaction between HRG and heparin (22). Aside from this, the relative levels of protein fucosylation and sialylation were found substantially changed between malignant and benign ovarian cancer patients (23).

Of particular interest, a prevalent genetic mutation in HRG has a profound effect on its glycosylation. The SNP rs9898, C to T with a population frequency of 49.14%, leads to an amino acid change at position 204 from Pro to Ser. This mutation changes the local sequence from Asn-Cys-Pro to Asn-Cys-Ser, leading to a new putative *N*-glycosylation site at Asn202 (following the *N*-glycosylation consensus sequence Asn-Xxx-Ser/Thr). Intriguingly, this *N*-glycosylation site was found to be associated with different diseases and afflictions. For example, pregnancy rates are diminished severely in women carrying homozygote Ser204 (24). Moreover, the rs9898 mutation may be associated with aging and risk of mortality (25). However, it remains unclear whether the *N*-glycosylation site introduced by rs9898/Pro204Ser is always occupied by an *N*-glycan, and what the functional consequences of this would be.

Here, we utilized LC–MS/MS-based (glyco)proteomics to characterize HRG from human plasma as well as that of five recombinant variants with specific mutations, including the aforementioned Pro204Ser mutation, as well as the Asn493Ile mutation. To enrich the protein efficiently, we made use of an optimized immobilized metal affinity chromatography (IMAC) purification protocol enabled by the histidine-rich regions of HRG. In doing so, we first revealed the glycosylation pattern of endogenous plasma HRG and the deviant glycosylation pattern of HRG produced in different host cells (Chinese hamster ovary [CHO] and human embryonic kidney 293 [HEK293] cells). We observed that the rs9898/Pro204Ser-specific Asn202 site was indeed highly occupied by complex-type *N*-glycans. We also observed thus far unreported *O*-glycosylation sites at HRG Thr273 and Thr274 that were more than 90% occupied in all HRG variants studied. Since the *O*-glycosylation sites were in close proximity to one of the proposed plasmin-cleavage sites (Lys275), we tested the functional consequences of the glycosylation by assessing the kinetics and specificity of plasmin-induced cleavage of HRG (16). This assay revealed that the degree of plasmin-induced cleavage was substantially influenced by sialylation, in particular that of the *O*-linked glycans, and that the Pro204Ser mutation also noticeably affected the plasmin-induced cleavage. Our report therefore reveals how intricate glycoproteogenomic features of an abundant plasma protein such as HRG may help to regulate its function *in vivo*.

## Results

We initially aimed to extensively profile the glycosylation profiles of HRG by using peptide-centric glycoproteomics. For this, we investigated six different samples of HRG, namely one endogenous sample originating from human plasma, one recombinant sample originating from CHO cells, and four HRG mutants expressed and purified from HEK293 cells. The recombinant samples had fixed mutations at sites 204 and 493, being Pro204/Asn493 for CHO-derived HRG, and Pro204/Asn493, Pro204/Ile493, Ser204/Ile493, and Ser204/Asn493 for the four HRG recombinant variants expressed in HEK293 cells. All HRG samples were purified by using IMAC-affinity resins, making use of the histidine-rich patches in HRG (19, 26). Following purification, we digested each sample using trypsin, followed by bottom–up glycoproteomics and analysis by LC–MS/MS. In the database search, we included all five most-abundant MAFs of HRG (Supplementary Data Excel file 1).

**Figure 1:**
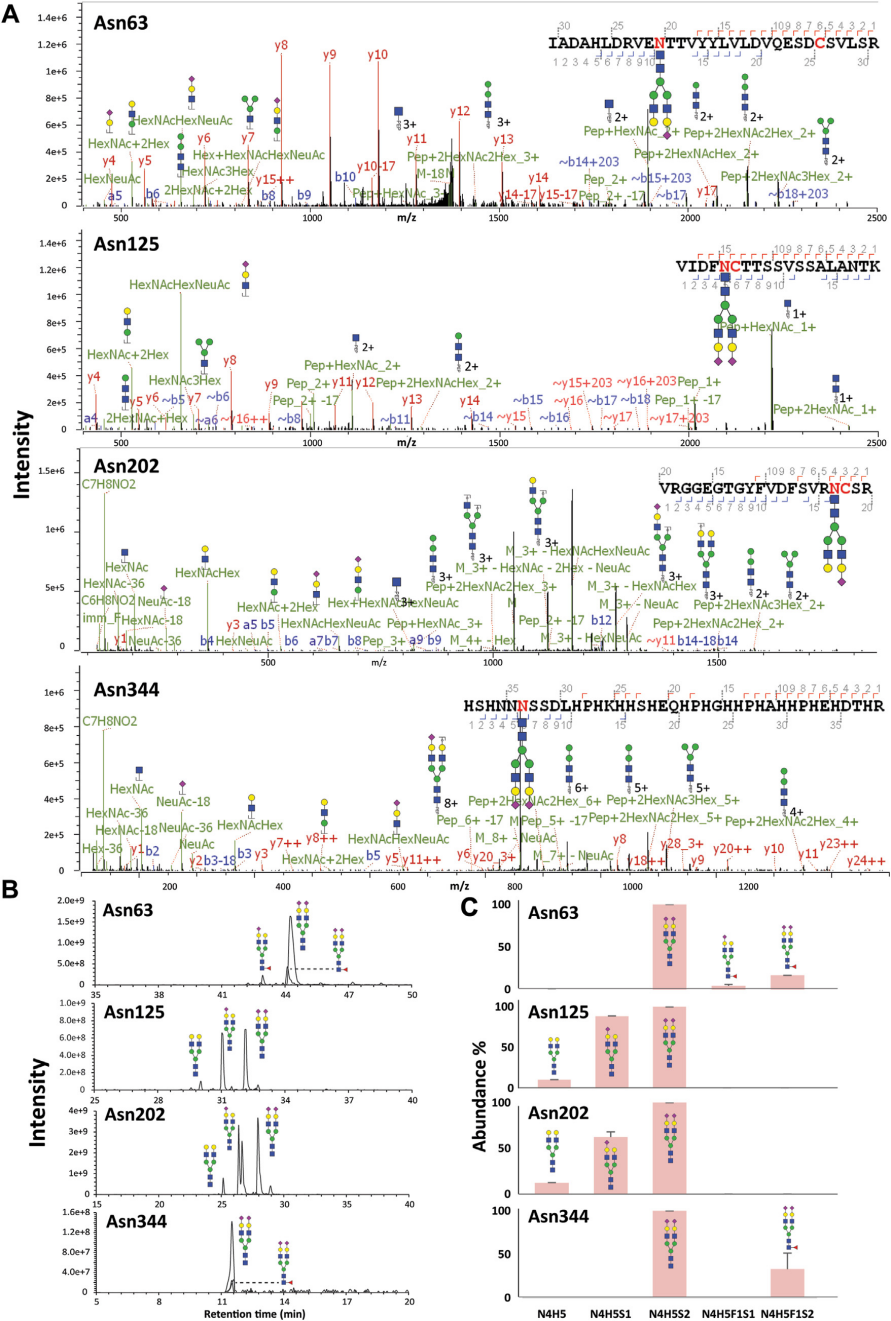
**Descriptive examples and characteristics of human plasma HRG *N*-glycosylation.***A*, illustrative MS/MS fragmentation spectra of glycopeptides covering each of the (potential) HRG *N*-glycosylation sites Asn63, Asn125, Asn202, and Asn344. These tryptic glycopeptides were fragmented by stepping HCD (sHCD). *B*, LC–MS traces of glycopeptides of each *N*-glycosite detected in HRG. *C*, abundance distribution of all glycans detected for each glycosylation site obtained by averaging PSM numbers across the different repetitive measurements. HRG, histidine-rich glycoprotein; PSM, peptide-spectrum match.

**Figure 2:**
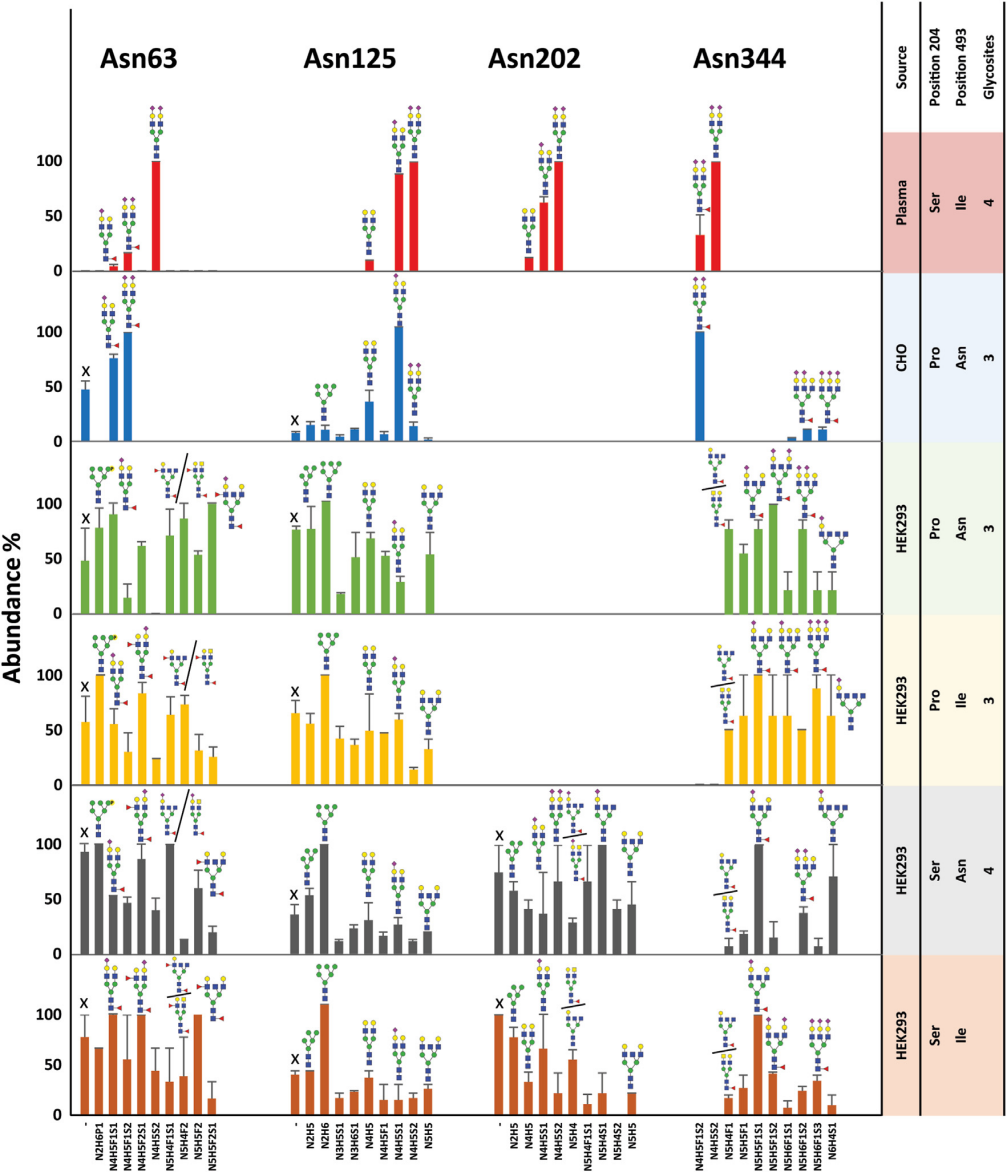
**Top 10 *N*-glycans detected for each *N*-glycosite on HRG, as purified from different sources.** The figures from *left* to *right* show the glycan characteristics on the glycosylation sites Asn63, Asn125, Asn202, and Asn344. The panels from *top* to *bottom* show the glycan characteristics for a single glycosylation site of plasma HRG, recombinant HRG produced in CHO cells, and recombinant HRG produced in HEK293 cells. X illustrates the *N*-glycosite is unoccupied. Glycan abundance for each glycosylation site was calculated by averaging PSMs from technical duplicates. The exact (mutant) amino acid present at site 204 and 493 in the different HRG samples is also annotated. Having a Pro204 erases the Asn202 glycosylation site. CHO, Chinese hamster ovary cell line; HEK293, human embryonic kidney 293 cell line; HRG, histidine-rich glycoprotein; PSM, peptide-spectrum match.

In doing so, we achieved an average sequence coverage of approximately 87%, which covers the targeted MAF-specific tryptic peptides containing position 204 and position 493, as well as glycopeptides harboring each reported *N*-glycosylation site. The bottom–up proteomics data validated that, as expected, the recombinant HRG variants correctly expressed the chosen genetic mutations and also revealed that the plasma-purified HRG harbored a Ser204 and an Ile493. We identified glycopeptides covering the *N*-glycosylation sites Asn63, Asn125, Asn202, and Asn344 (Fig. 1*A*) and quantified their relative abundances across all studied HRG samples using glyco-peptide-spectrum match (PSM) counts, averaging over technical replicates (Figs. 1*C* and 2).

### HRG *N*-glycosylation profiling

The mass spectrometric data, particularly the fragmentation evidence, revealed the presence of a broad variety of glycan features across all HRG samples and sites: oligomannose, hybrid and complex-type species, core and antennary fucosylation, general diantennary and triantennary, as well as lower-abundant features, such as phosphomannosylation and LacDiNAc antennae (Figs. S1–S8).

For quantifying glycan distributions at specific glycosylation sites, researchers may perform manual integration of MS1 areas for individual glycopeptides using tools like Skyline (MacCoss Lab Software) (27). In our current study, however, this process of manual peak selection and integration proved laborious and error prone, especially for highly heterogenous glycosylation from HRG from HEK293 cell lines, covering more than 100 distinct glycans across multiple (miscleaved) peptides (Supplementary Data Excel file 2). One viable alternative for quantification involves counting PSMs (annotated MS2 spectra), an approach that is commonly used for high-throughput studies and those with complex samples (27, 28, 29). The expectation is that higher-abundant analytes exhibit longer elution times, while showing more detectable charge states, more structural isomers, and more peptide miscleavages. Consequently, this leads to a higher chance of triggering an MS2 scan as well as a higher chance of identification of that scan, both resulting in a larger number of PSMs for analytes with higher abundance. Making use of this approach in our study, we acquired the abundance of each glycan by averaging glyco-PSM detections between the higher-energy collisional dissociation (HCD) and stepping HCD (sHCD) fragmentation methods (Supplementary Data Excel file 2).

Human plasma endogenous HRG *N*-linked glycosylation sites were found to be fully occupied with diantennary complex–type glycans (Figs. 1, *B* and *C* and 2). For all the *N*-glycosylated sites, Asn63, Asn125, Asn202, and Asn344, disialylated diantennary species N4H5S2 was always dominant in the plasma HRG, whose relative abundance was set as 100%. Monosialylated N4H5S1 was also abundant on Asn125 and Asn202, with relative intensities of 88% and 62% compared with N4H5S2. Next to these major glycan features, additional nonsialylated and fucosylated diantennary glycans could be detected with relative intensities ranging from 10% to 33% (Figs. 1, *B* and *C* and 2).

The glycan patterns of recombinant HRG from CHO cells were found to be more complex, with more variation in glycan types and higher levels of fucosylation. The most abundant glycans on sites Asn63 and Asn344 were sialylated diantennary glycans, carrying also fucosylation (N4H5F1S2). It is worth noting that Asn63 was not fully occupied, with 48% unoccupancy compared with the abundance of N4H5F1S2. By comparison, the sialylated diantennary glycans with fucosylation on position Asn125 were not in the top 10 glycans, the dominant glycan instead being the monosialylated diantennary species N4H5S1. No glycosylation could be detected on Asn202, coinciding with the lack of an *N*-glycosylation motif because of the presence of Pro204 (Fig. 2).

The four recombinant HRG variants, expressed in and purified from HEK293 cells, covering the four different combinations of the mutations on positions 204 and 493, showed a wide range of *N*-glycosylation types, including oligomannose, phosphomannose, hybrid and triantennary complex–type species, as well as features such as antennary fucosylation and LacDiNAcs, all these being mostly absent in plasma HRG (Figs. S1–S8). For Asn63, sialylated complex glycans with fucosylation (N5H5F2S1) and phosphorylated oligomannose species (N2H6P1) were found to be most abundant. This Asn163 site was also found to be to a substantial amount unoccupied. Oligomannose glycan N2H6 was the most abundant glycan detected on Asn125 in each of the four recombinant HRG from samples expressed in HEK293 cells, whereas the most abundant glycan on Asn344 was found to be either N5H5F1S1 or N5H5F1S2. The glycosylation site Asn202, which only occurs following the Pro204Ser mutation, was found to be nearly fully occupied, ranging from 90% to 93% (Fig. 2). For glycan compositions N5H4F1, N5H4F2, N5H4, N5H4S1, and N5H4S2, all detected in HEK293-produced recombinant HRG, both LacdiNAc motifs and antennary GlcNAcs were likely options based on the fragmentation patterns (Figs. S5 and S8). At the same time, no evidence was observed that would support the presence of bisecting GlcNAcs.

### Discovery of HRG *O*-glycosylation sites

**Figure 3:**
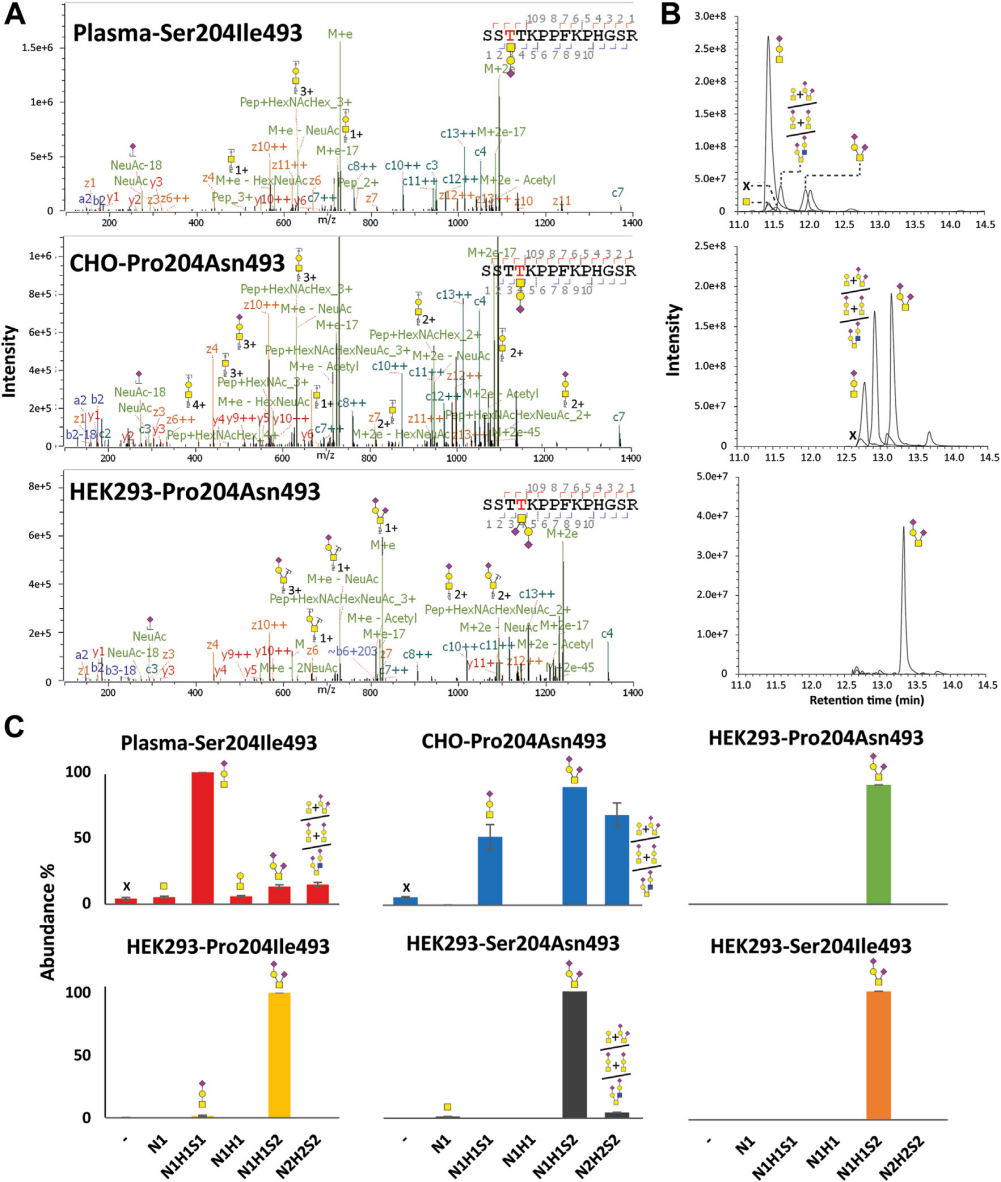
***O*-Glycan profiling of HRG variants.***A*, illustrative EThcD MS/MS spectra of tryptic peptides harboring *O*-glycans. From *top* to *bottom* are depicted the tryptic peptides from plasma HRG, HRG from CHO cells, HRG from HEK293 cells (mutations Pro204/Asn493), respectively. *B*, prototypical LC–MS traces of the *O*-glycopeptides detected for HRGs. *C*, distribution of the detected *O*-glycans in each sample. The *O*-glycan abundances were calculated by averaging MS1 peak areas. X illustrates the *N*-glycosite is unoccupied. CHO, Chinese hamster ovary cell line; EThcD, electron transfer/HCD; HEK293, human embryonic kidney 293 cell line; HRG, histidine-rich glycoprotein.

Next to the well-known HRG *N*-glycosylation, we found strong evidence for *O*-glycosylation in each of the investigated HRG samples (Supplementary Data Excel file 3). To obtain additional proof for this novel feature, we carefully inspected the electron transfer/HCD (EThcD) fragmentation data, which are typically more beneficial for the analysis of *O*-glycosylation than HCD and sHCD would be (30, 31). EThcD can cleave the peptide backbone without dissociating the glycan, which is beneficial for *O*-glycan site localization. For example, in the EThcD fragmentation data of endogenous plasma HRG, we found spectra with glycan-specific oxonium ions and mass differences between fragment ions hinting at *O*-glycans. A mass difference of 757.2718 Da between the z11^2+^ and z12^2+^ ions was observed, which we assigned to N1H1S1 (656.2276 Da) on position Thr273 (101.0477 Da) (Fig. 3*A*). Also, in the recombinant HRG samples produced from CHO cell lines, we could observe similar mass differences of 757.2488 Da between the z10^2+^ and z11^2+^ ions, indicating an N1H1S1 composition on Thr274, whereas for HEK293 cell–derived HRG, a mass difference of 1048.4062 Da could be observed between the c3^+^ and c4^+^ ions, indicating a Thr274 with an attached N1H1S2 (947.3230 Da) (Fig. 3*A*). These illustrative examples were further corroborated by the same peptides also harboring other well-known *O*-glycan compositions.

**Figure 4:**
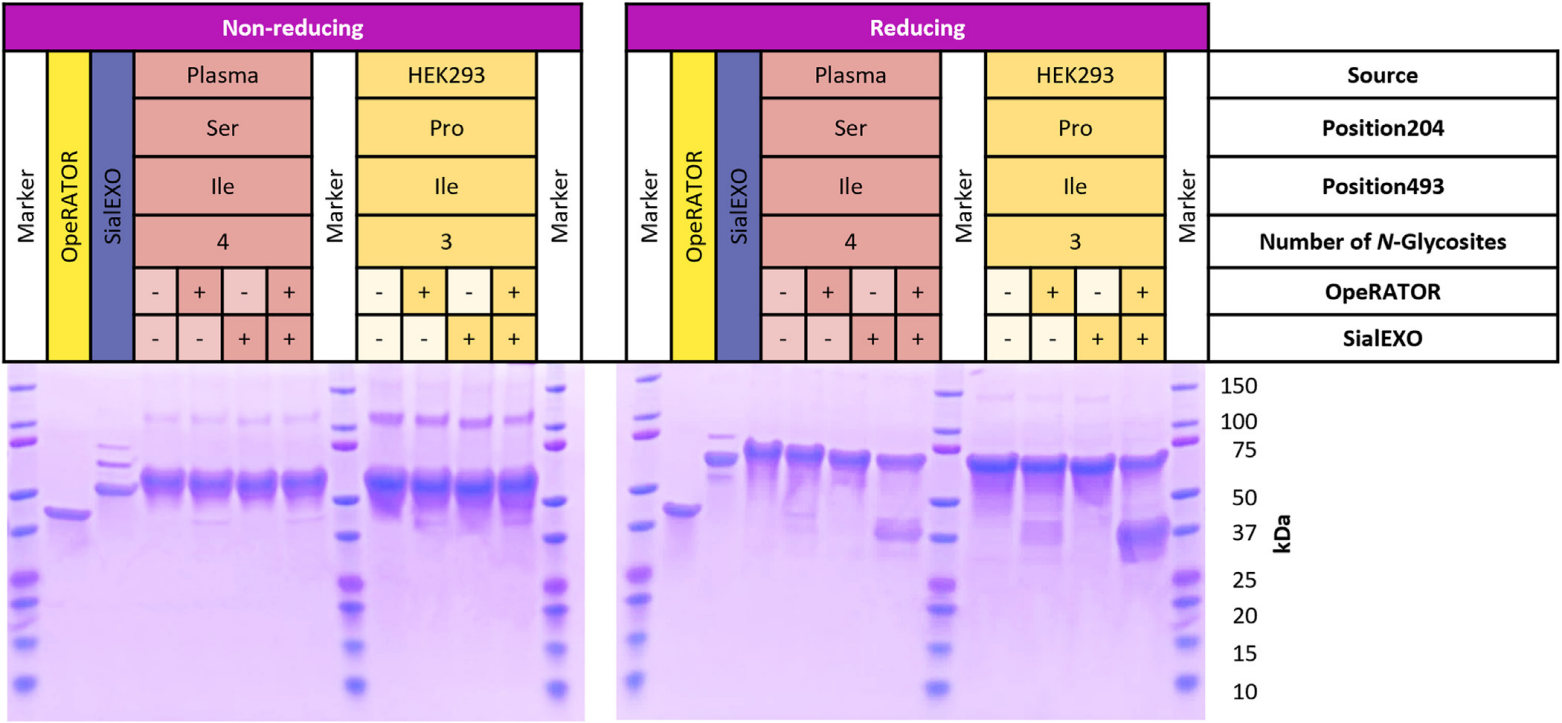
**Nonreducing and reducing gels of HRG samples incubated with ± OpeRATOR and ± SialEXO.** The HRG sample variants are from plasma (*red*) and HEK293 cell lines (genotype Pro204/Ile493, *yellow*). For each sample, four experimental conditions were used, either no treatment, incubation with OpeRATOR, incubation with SialEXO, or incubation with OpeRATOR and SialEXO combined. OpeRATOR and SialEXO were also loaded separately. To make the bands stand out from the background in the figures, the contrast was set to 45% and the brightness of the gel was set to 15%. HEK293, human embryonic kidney 293 cell line; HRG, histidine-rich glycoprotein.

To further substantiate the existence of these newly discovered HRG *O*-glycosylation sites, we used the OpeRATOR enzyme to digest the HRG proteins and analyzed the protein fragments by SDS-PAGE. OpeRATOR is a very specific protease that can digest core 1 *O*-glycoproteins and peptides N-terminally of *O*-glycosylation occurring on Ser residues or Thr residues. OpeRATOR is most effective toward sites with desialylated *O*-glycans; therefore, we also used SialEXO to remove the sialic acids on the glycans prior to digestion by OpeRATOR (32). In theory, after digesting with OpeRATOR and SialEXO, two fragments will be generated with masses of approximately 31 kDa and 36 kDa, separated by the *O*-glycosylation site on Thr273/274. As such, we treated HRGs under four experimental conditions: no treatment, incubation with SialEXO, incubation with OpeRATOR, and combined incubation with SialEXO and OpeRATOR. Since there are several disulfide bonds connecting the cleaved peptides, we ran both nonreducing and reducing SDS-PAGE gels. As expected, the nonreducing gels did not provide information on the cleavage, but they did show intact glycosylated HRG (∼65–67 kDa), potential HRG dimers at 120 to 135 kDa, OpeRATOR (42 kDa), and SialEXO (66 kDa, but migrating lower than HRG). Within the same experiment, the reducing gel showed broad bands appearing in the ∼31 to 36 kDa range in both conditions with OpeRATOR enzyme but much more pronounced in the condition with additional desialylation. Therefore, both the gel- and proteomics-based results were strongly indicative of a highly occupied and sialylated *O*-glycosylation site within HRG (Fig. 4).

To distinguish the *O*-glycosylation site localizations of human endogenous HRG and recombinant HRGs from CHO and HEK293 cell lines, we used OpeRATOR and SialEXO to digest HRG, performed in-solution digestion with trypsin, and then studied the resulting peptides by bottom–up LC–MS/MS. After treatment, we could see that the glycans among different cell lines were N1H1 (possibly from N1H1, N1H1S1, and N1H1S2) as well as N2H2 (possibly from N2H2, N2H2S1, and N2H2S2). Quantifying the *O*-glycan positions *via* the MS1 peak area of the most abundant detected glycopeptides, we could find that *O*-glycosylation can be situated on both Thr273 and Thr274 among all samples (Fig. S9, Supplementary Data Excel file 4). For plasma HRG, modified with N1H1, peptides were mainly cleaved at the N terminus of Thr274 (74%), the remainder being cleaved at the Thr273 position. For both recombinant HRGs produced in CHO and HEK293 cell lines, the percentage of cleavage was similar between sites Thr273 and Thr274, on average 53% and 50% of being cleaved at the Thr273 position, respectively.

### HRG *O*-glycosylation profiling

To obtain the *O*-glycan profiles on Thr273/Thr274, we integrated the MS1 areas of the most frequently detected *O*-glycopeptides (Fig. 3, *B* and *C*, Supplementary Data Excel file 5). The peptides proved to be highly occupied with *O*-glycosylation in all six studied HRG samples, the occupancy ranging from 93% to 100%. The *O*-glycans were found to be highly sialylated, endogenous plasma HRG being predominantly monosialylated, that is, N1H1S1 (100%). Recombinantly produced HRG variants displayed again different glycosylation characteristics compared with the endogenous variant. Both CHO cell– and HEK293 cell–derived HRG variants had disialylated *O*-glycans (N1H1S2) as their most abundant feature (100%). CHO-derived HRG in addition revealed abundant *O*-glycan composition N1H1S1 with 58% and larger *O*-glycan composition N2H2S2 with 76% (Supplementary Data Excel file 5). As information from MS2 fragmentation was scarce, we note that N2H2S2 could potentially originate from two glycans on both Thr273 and Thr274, such as two N1H1S1 on both sites, or N1H1 and N1H1S2 on each site.

### Mutation and glycosylation affect plasmin-induced cleavage in HRG

We characterized here in detail the glycosylation and mutational features of six different HRG samples, purified from different sources. We found that these HRG variants displayed quite variable glycoproteogenomic features. Evidently, an important issue to address is whether these features affect the structure and function of HRG. It has been reported that the biological activity of HRG *in vivo* may be regulated *via* plasmin-mediated proteolytic cleavage (16). Therefore, we set out to study the plasmin-induced cleavage of HRG *in vitro*, particularly that of the different HRG mutants and glycoproteoforms, focusing on the kinetics and specifics of the formed protein fragments. There are four putative plasmin-induced cleavage sites in HRG, namely at Lys275, Lys291, Arg339, and Arg439 (16). Among these, the novel *O*-glycosylation sites Thr273/274 are in very close proximity to Lys275, which we hypothesized might therefore influence the plasmin cleavage kinetics and specificity at this site.

In our assays, we incubated HRG variants with 0%, 3%, or 30% plasmin (w/w) and ran SDS-PAGE gels to observe cleavage products for 1 h (Fig. S10, *A* and *B*). We also introduced SialEXO to investigate whether sialyation of HRG influenced plasmin-induced cleavage. As expected, without adding plasmin, no clear cleavage products were observed, except for HRG produced in and purified from CHO cells. In that case, the observed protein fragments were likely already copurified during the recombinant production process. Incubation with 3% and 30% plasmin (for 1 h) clearly induced the formation of several cleavage products with lower masses for all studied HRG variants, exposing also quite a few different patterns (Fig. S10, *A* and *B*).

**Figure 5:**
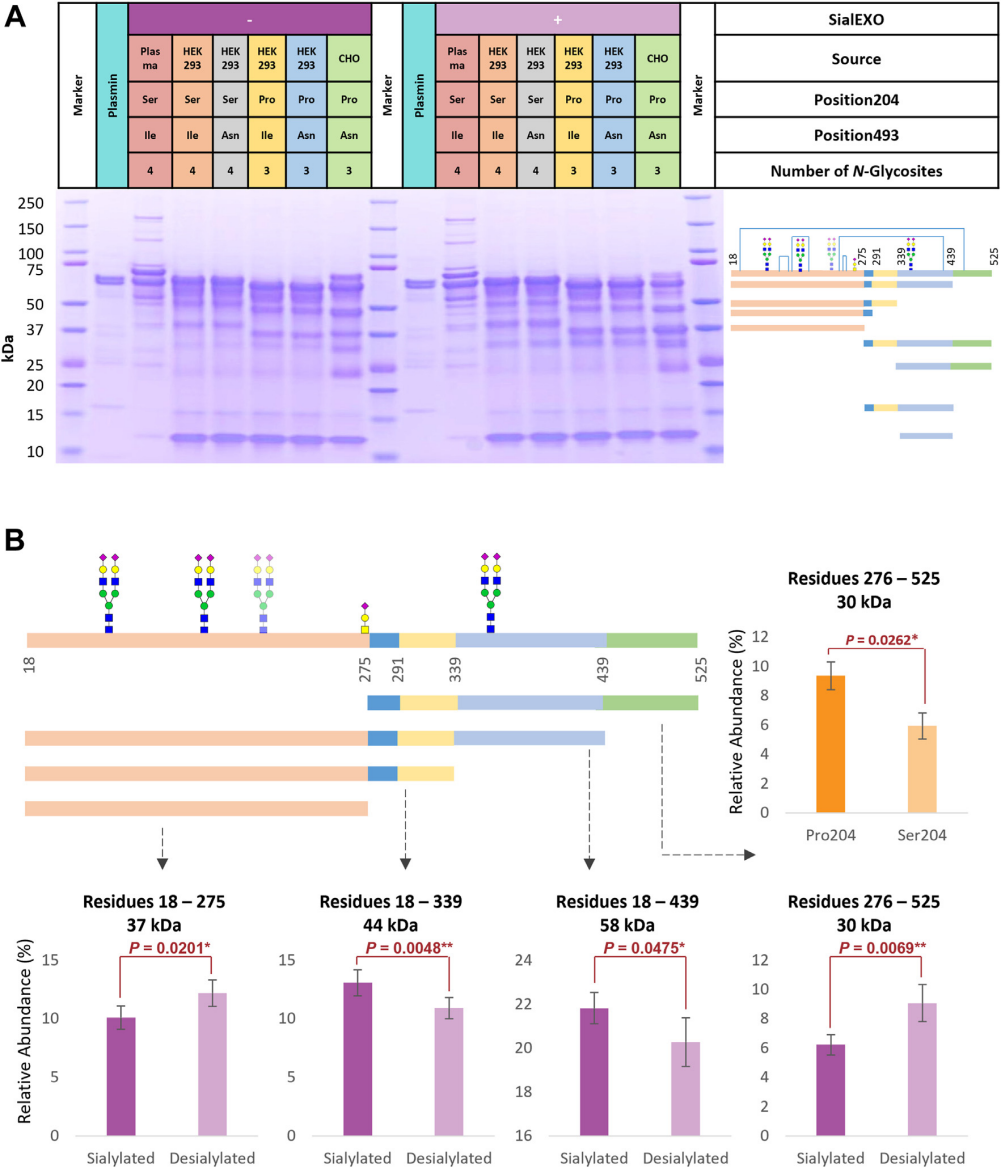
**Plasmin-induced cleavage of HRG.***A*, reducing gel of different HRG samples treated with 3% plasmin for 1 h. The lanes from *left* to *right* show plasmin only, HRG from plasma incubated with plasmin, HRG from HEK293 cells harboring the distinctive allotype mutations (Ser204/Ile493, Ser204/Asn493, Pro204/Ile493, and Pro204/Asn493), and HRG originating from CHO cell lines ± incubation with SialEXO treatment. The schematic figures in the *left panel* of the gel indicate the protein fragment assignments. HRG fragments of different colors represent the segments that can be generated by plasmin, particularly because of C-terminal cleavage at sites Lys275, Lys291, Arg339, and Arg439 (16). The displayed glycosylation is the most abundant species on endogenous plasma HRG, *blue lines* represent disulfide bonds. To make the bands stand out from the gel background in the figures, the contrast value was set to 45% and the brightness of the gel was set to 15%. *B*, bar chart of HRG cleavage products with significantly different relative abundances, linked to either the Pro204Ser mutation and/or sialylation. Independent (unpaired) sample *t* tests were used to determine whether the means of fragments from the samples of HRG with Pro204 and the ones of HRG with Ser204 were significantly different from each other (n_1_ = 6, n_2_ = 6). Paired sample *t* tests were used to determine whether the means of segments from the sialylated samples and desialylated groups were significantly different from each other (n = 6). All the related data are shown in Supplementary Data Excel file 6. CHO, Chinese hamster ovary cell line; HEK293, human embryonic kidney 293 cell line; HRG, histidine-rich glycoprotein.

We selected the incubation with 3% plasmin for a more detailed comparison among the HRG variants. Following incubation, HRG fragments of approximately 67, 58, 44, 39, 37, 30, 23, 14, and 12 kDa started to appear. The bands were assigned (based on the theoretical masses) to different reported cleavage products (Fig. 5*A*, Supplementary Data Excel file 6) (16), of which we achieved mean abundance values by integrating the bands. This revealed several differences directly. For instance, the abundance of the 30 kDa bands (residues 276–525) from HRG variants with either Pro204 or Ser204 differed by 1.6-fold (9% SE ± 1% *versus* 6% ± 1%, *p* = 0.0262), illustrating that Pro204Ser substitution hampers the plasmin-induced cleavage of HRG at site Lys275 *in vitro*. Furthermore, for bands with an apparent mass ∼58 kDa (residues 18–439), the abundance was higher for sialylated HRG compared with desialylated HRG (22% ± 1% *versus* 20% ± 1%, *p* = 0.0475). For the 44 kDa bands (residues 18–339), the fragments originating from sialylated HRG were also more abundant than for desialylated HRG (13% ± 1% *versus* 11% ± 1%, *p* = 0.0048). The abundance of the segments of 37 kDa (residues 18–275) was lower for sialylated HRG variants when compared with desialylated HRG variants (10% ± 1% *versus* 12% ± 1%, *p* = 0.020). Finally, the fragments around 30 kDa (residues 276–525) were less abundant for sialylated HRGs than for desialylated HRG (6% ± 1% *versus* 9% ± 1%, *p* = 0.0069). All these data suggest that both specific mutations as well as glycosylation influence the susceptibility of HRG to plasmin cleavage (Supplementary Data Excel file 6).

## Discussion

HRG is an abundant protein in human plasma, which has been proposed as a potential biomarker for diagnosing and predicting prognosis of sepsis (33), breast cancer (34), ventilator-associated pneumonia (35), pulmonary fibrosis (36), and coronavirus disease 2019 (37, 38). In this study, we investigated especially the glycosylation characteristics of distinct HRG variants. These were purified from or expressed in different systems and displayed a large variety in glycan characteristics when analyzed by MS-based glycoproteomics. Endogenous HRG purified from plasma seemingly exhibited the simplest glycosylation pattern, with predominantly N4H5S2 glycans attached to all *N*-glycosylated sites. Recombinant HRG variants (both from CHO cells or HEK293 cells) displayed more variable and complicated glycan profiles. At the *N*-glycosylation site Asn202, induced by the SNP rs9898, we detected in the endogenous plasma HRG sample a glycosylation pattern quite similar to that found at the other sites, namely with dominant N4H5S2 and N4H5S1.

As protein glycosylation involves a wide range of enzymes, it is not unexpected that different sources show distinct glycosylation profiles for the same protein. These differences are likely because of the host expression system, which, for example, can differ in glycosyltransferase abundance (39). Compared with natural HRG, the *N*-glycosylation of the recombinant HRG from CHO cells was found to be only partially sialylated but in sharp contrast also highly fucosylated. The different abundance of sialic acids might be attributed to the CHO cell line lacking α2,6-sialyltransferase (St6gal1) so that the linkage of sialic acid of protein derived from CHO cells can only be α2,3 (40, 41). The absence of this enzyme may result in the lower abundance of sialylation.

For the HRG expressed in HEK293 cells, the glycan profile was more widely distributed. Not only did the levels of glycan fucosylation and sialylation vary strongly across the different glycosylation sites, larger antennarities and oligomannose glycan species were observed as well. Next to this, phosphomannose, hybrid species, antennary fucosylation, and LacDiNAc motifs were detected in HRG from HEK293 cells but were absent in endogenous HRG. These features are more frequently observed in recombinant proteins from HEK293 cell lines, examples being fetuin-A, erythropoietin, and factor VIII (42, 43, 44), and are likely caused by the expression system. However, our observations highlight the stark contrast between the glycosylation of endogenous HRG and its variants produced in cell lines, with functional consequences.

Importantly, we describe here two novel *O*-glycosylation sites within HRG, at Thr273 and Thr274, both being substantially occupied. *O*-Glycosylation sites in plasma-derived HRG were mainly of the N1H1S1 type, whereas the recombinant HRG variants were occupied mostly with N1H1S2. To confirm the co-occurrence of the two adjacent *O*-glycosylation sites, we used the enzymes OpeRATOR and SialEXO. OpeRATOR is an *O*-glycan-specific protease that digests proteins N-terminally of glycosylated (core 1) Ser and Thr residues, a process that is more efficient when the sialic acids are being removed first (32). In doing so, we found that the *O*-glycosylation could be situated at either position Thr273 and/or Thr274, with the glycans N1H1 and N2H2 dominant after removing the sialic acids. It has been shown that protein *O*-glycosylation can play a crucial role in various infectious diseases and can significantly influence the outcome of the immune response and may be an important factor in cancer progression as well (45). Therefore, these newly discovered *O*-glycosylation sites need to be taken into account when investigating the characteristics and function of HRG.

Although the histidine-rich region of HRG released by plasmin cleavage has been reported to play a role in mediating the antiangiogenic and possibly antimicrobial effects of the molecule (16), the specific role of HRG glycosylation in this process is unknown. Seeking to fill this gap, we found that (1) the sialylation of the *O*-glycan on Thr273/274, (2) the sialylation of *N*-glycosylation, as well as (3) the allotype-dependent *N*-glycosylation on Asn202 all had an effect on the kinetics and specificity of plasmin-induced cleavage of HRG. The effect of the *O*-glycosylation may be easiest to explain, as the sites Thr273/274 are in very close proximity to known plasmin cleavage site Lys275 (16). The additional *N*-glycosylation site on Asn202, highly occupied with a large diantennary *N*-glycan (N4H5S2), might not directly be in contact with plasmin cleavage sites but could affect the overall conformation of HRG to such a degree that the interaction between plasmin and HRG becomes affected. Protein glycosylation can affect proteolysis of surrounding region, as steric hindrance and charge effects may inhibit or promote digestion (46). One proposed function of protein *O*-glycosylation is protection against proteolytic degradation (47), so our new finding that HRG is also an *O*-glycoprotein is important in this context. Interestingly, endogenous HRG proved more resistant to plasmin-induced cleavage than recombinant HRG variants *in vitro*, making it likely that additional structural differences exist among these HRG variants.

## Experimental procedures

### Chemicals and reagents

Human plasma containing EDTA from a single healthy individual was obtained from Zen-Bio. One recombinant HRG variant, containing the mutations Pro204 and Asn493, was produced in CHO cell lines by PeproTech. Four other HRG variants, with pairwise combinations of targeted mutations for positions 204 and 493, were produced in HEK293E-253 cells (HEK293) by ImmunoPrecise. HRGs were expressed with sequences matching allelic variants on position 204 or 493 in human population: Pro204/Asn493, Pro204/Ile493, Ser204/Ile493, and Ser204/Asn493.

Cobalt-loaded resin was acquired from Thermo Scientific. Imidazole was purchased from Sigma–Aldrich. Tris–HCl, Tris(2-carboxyethyl)phosphine, chloroacetamide, sodium deoxycholate (SDC), and TFA were purchased from Sigma–Aldrich. Oasis HLB 96-well μElution Plates were purchased from Waters. Sequencing-grade trypsin was obtained from Promega. SialEXO and OpeRATOR were obtained from Genovis. Plasmin was purchased from Abcam. Formic acid (FA) and acetonitrile were acquired from Biosolve Chimie. The SDS-PAGE standards and Imperial Protein Stain were purchased from Bio-Rad and Thermo Scientific, respectively.

### Plasma HRG protein purification

HRG was isolated from human plasma using IMAC using a cobalt-loaded resin, as previously described (19). Briefly, after incubating plasma and cobalt-loaded resin in binding buffer (25 mM Hepes, 500 mM NaCl, pH 7.8) for 3 h at 4 °C, we used washing buffers (25 mM Hepes, 500 mM NaCl, pH 7.8) containing concentrations of imidazole ranging from 15 mM to 40 mM to wash the beads. After that, the purified HRG was eluted with eluting buffer (500 mM imidazole, 25 mM Hepes, 500 mM NaCl, pH 7.8).

### In-solution digestion

Purified HRG was resuspended in 100 mM Tris–HCl and then mixed with digestion buffer containing 200 mM Tris–HCl (pH 8.5), 2% w/v SDC, 10 mM TCEP, and 60 mM chloroacetamide. The sample was denatured at 95 °C for 10 min and then incubated in the dark for 45 min. The resulting peptide mixtures were digested overnight at 37 °C by trypsin (1:10; w/w). The next day, SDC was removed *via* acid precipitation (0.5% TFA). The peptides were desalted by using an Oasis HLB 96-well μElution Plate, then dried by vacuum centrifugation, and stored at −80 °C.

### LC–MS/MS

Shotgun LC–MS/MS was performed by means of an UltiMate 3000 UHPLC system (Thermo Fisher Scientific) coupled to an Orbitrap Fusion Mass Spectrometer or an Orbitrap Fusion Lumos Mass Spectrometer (Thermo Fisher Scientific). A total of 250 ng of the digested peptides were loaded on a trap column (PepMap Neo Trap Cartridge, 174500, 300 μm diameter) (Thermo Fisher Scientific) coupled to a 50 μm inner diameter 50 cm analytical column (in-house packed with ReproSil-Pur C18-AQ, 2.4 μm diameter) (Dr Maisch GmbH). As for gradient separation, 0.1% FA (v/v) was used as mobile phase A, whereas 0.1% FA in acetonitrile (v/v) was used as mobile phase B. At the first stage, the mobile phase increased from 9% to 13% for 1 min, rising from 13% to 44% in the next 40 min, then it rose from 44% to 99% for 3 min, and maintained 99% for 4 min. Then it decreased to 9% in 1 min and maintained 9% for 10 min. The flow rate was set as 300 nl/min. Peptides were ionized using a spray voltage of 2 kV in combination with the ion transfer capillary at 275 °C. The mass spectrometer was set to acquire full-scan MS spectra (*m/z* 350–2000) for a maximum injection time of 50 ms at a mass resolution of 120,000. For HCD fragmentation, MS/MS acquisition was performed in the HCD cell. For this, the readout was in the Orbitrap mass analyzer at a resolution of 60,000 from *m/z* 120 to 4000, whereas the maximum injection time was 50 ms and the normalized collision energy was 29%. For HCD product–dependent sHCD and EThcD fragmentation, triggering occurred when at least three of the following ions were detected with a mass error ≤20 ppm within the preceding HCD scan: 127.039, 145.0495, 163.0601, 243.0264, 405.0793, 138.0550, 168.0655, 186.0761, 204.0867, 274.0921, 292.1027, 366.1395, 407.1660, 512.1974, and 657.2349 Da. sHCD was recorded with a resolution of 60,000 from *m/z* 120 to 4000. The collision energies were set as 10%, 25%, and 40%, with a maximum injection time of 150 ms. EThcD (15% supplemental activation) was recorded with a resolution of 60,000 from *m/z* 120 to 4000 and a maximum injection time of 150 ms as well.

### Database search and data analysis

Raw files were analyzed using Byonic v4.3.4 (Protein Metrics). MS/MS spectra were searched against the human database (Swiss-Prot database, release date July 2021, 20,398 entries), supplemented with HRG sequences carrying five of the dominant mutations with MAFs more than 10% (Supplementary Data Excel file 1). The search parameters were as follows: fixed modification of cysteine residues (+57.0215 Da), variable common modification of *N*-glycans and *O*-glycans, variable rare modification of methionine oxidation (+15.9949 Da), phosphorylation (+79.9663 Da), glutamine to pyroglutamate conversion (−17.0265 Da), glutamic acid to pyroglutamate conversion (−18.0106 Da), and specific trypsin cleavage were set as variable modifications with a total common maximum of 1, rare maximum of 1, at most three missed tryptic cleavage sites, 10 ppm error tolerance in MS, and 20 ppm error tolerance in MS/MS. The glycans used for the database search are listed in Supplementary Data Excel file 7. PSM number was used to perform a relative *N*-glycan quantification of the glycan features. For curation of the Byonic PSMs, we made use of a score threshold of 150 and |Log Prob| threshold of 2, as well as controlling the false discovery rate to be less than 0.01 (Supplementary Data Excel file 2). The relative quantification of each glycan was achieved by averaging the PSM number of each glycopeptide with both HCD and sHCD fragmentation methods for *N*-glycosylation. For normalization, the abundance of the glycan with the highest average abundance was set as 100%, the others expressed as a fraction thereof.

Skyline (version 3.7.0.11317) was used to perform a relative glycan quantification of the *O*-glycan features as well as a relative *O*-glycan localization quantification from the MS level (Supplementary Data Excel files 4 and 5). To achieve this, each of the typical glycopeptides found with Byonic was integrated separately (Supplementary Data Excel file 3). The most frequently detected peptide sequences with *O*-glycosites are shown in Supplementary Data Excel file 8. The integrations acquired in this manner were then reviewed to meet the following standards: (1) an error of ≤5 ppm to the theoretical mass; (2) exhibiting an idotp value ≥0.80 when compared with the theoretical isotopic pattern; (3) elution time falling within a range of ±2 min around the average retention time for the corresponding peptide; and (4) no observable instances of overlapping isotopic patterns. The abundance of peak area of each glycopeptide was then exported and used for relative quantification. Even though HCD, sHCD, and EThcD fragmentation had different MS/MS methods, the MS method was the same. Therefore, the relative quantification of each glycan was achieved by averaging the peak areas of each glycopeptide MS spectra with HCD, sHCD, and EThcD fragmentation methods for *O*-glycosylation (Supplementary Data Excel file 5). For normalization, the abundance of the glycan with the highest average abundance was set as 100%, the others expressed as a fraction thereof.

### OpeRATOR and plasmin digestion

OpeRATOR and SialEXO were first reconstituted in ddH_2_O each to a concentration of 40 units/μl. A total of 3 μg HRG was then treated with three units of SialEXO and three units of OpeRATOR for 2 h at 37 °C. Single-treated HRG (with either OpeRATOR or SialEXO) and untreated HRG were used as negative controls.

For generating plasmin-cleaved HRG, 3 μg of HRG was incubated with 0%, 3%, or 30% (w/w, 0, 0.09, and 0.9 μg) plasmin and diluted to 10 μl with ddH_2_O at 37 °C for 1 h. To desialylate HRG, we treated samples with 1 unit per 1 μg SialEXO for 2 h at 37 °C before the plasmin treatment.

### SDS-PAGE

Polyacrylamide gel electrophoresis was used to visualize protein cleavage. For this, a total of 3 μg sample was mixed with loading buffer and then heated to 95 °C for 5 min, before being loaded on SDS-PAGE gels. Electrophoresis was carried out at 160 mA for 1 to 2 h until the molecular weight standards properly separated. The gel was then removed and incubated in Imperial Protein Stain solution at room temperature for 1 h.

For the gels of plasmin-cleaved HRGs, we interpreted each band according to the apparent molecular masses and corresponding theoretical fragment masses based on the mass of peptide sequences and modifications (Supplementary Data Excel file 6) (16).

*Gray values* of each band were determined with ImageJ (v1.53t; the National Institutes of Health) with the following steps: (1) setting image type as 8 bit, (2) subtracting the gray background, (3) selecting and plot the lanes, and (4) measuring the area of the lanes. Then we calculated the relative abundance of each interpreted band for each sample. Independent (unpaired) sample *t* tests and paired sample *t* tests were used to determine the significance of differences between the bands from different plasmin-cleaved HRG samples. Independent-sample *t* tests were used to compare the band abundances of groups from the samples of HRG with Ser204 and the ones of HRG with Pro204 (n_1_ = 6, n_2_ = 6). Paired sample *t* tests were used to compare the band abundances of the sialylated samples and desialylated groups (n = 6) (Supplementary Data Excel file 6).

### Visualization of glycans

Structural schemes are given in terms of *yellow circle* (galactose, H), *red triangle* (fucose, F), *blue square* (*N*-acetylglucosamine, N), *yellow square* (*N*-acetylgalactosamine, N), *dark magenta diamond* (*N*-acetylneuraminic acid, S), *green circle* (mannose, H), and *yellow circle* with capital letter P (phosphate, P). We followed the CFG standard and drew the glycan cartoons with Glycoworkbench 2.1 (build 146) (48, 49).

## Data availability

The mass spectrometry proteomics data have been deposited to the ProteomeXchange Consortium *via* the PRIDE partner repository with the dataset identifier PXD044353 (50).

## Supporting information

This article contains supporting information.

## Conflict of interest

The authors declare that they have no conflicts of interest with the contents of this article.

## Supporting information

Supplementary Data Excel File 1

Supplementary Data Excel File 2

Supplementary Data Excel File 3

Supplementary Data Excel File 4

Supplementary Data Excel File 5

Supplementary Data Excel File 6

Supplementary Data Excel File 7

Supplementary Data Excel File 8

## References

[1] Poon I.K.H., Patel K.K., Davis D.S., Parish C.R., Hulett M.D. (2011). Histidine-rich glycoprotein: the Swiss Army knife of mammalian plasma. Blood.

[2] MacQuarrie J.L., Stafford A.R., Yau J.W., Leslie B.A., Vu T.T., Fredenburgh J.C. (2011). Histidine-rich glycoprotein binds factor XIIa with high affinity and inhibits contact-initiated coagulation. Blood.

[3] Lijnen H.R., Hoylaerts M., Collen D. (1980). Isolation and characterization of a human plasma protein with affinity for the lysine binding sites in plasminogen. Role in the regulation of fibrinolysis and identification as histidine-rich glycoprotein. J. Biol. Chem..

[4] Gorgani N.N., Parish C.R., Easterbrook Smith S.B., Altin J.G. (1997). Histidine-rich glycoprotein binds to human IgG and C1q and inhibits the formation of insoluble immune complexes. Biochemistry.

[5] Poon I.K.H., Hulett M.D., Parish C.R. (2010). Histidine-rich glycoprotein is a novel plasma pattern recognition molecule that recruits IgG to facilitate necrotic cell clearance via FcgammaRI on phagocytes. Blood.

[6] Heimburger N., Haupt H., Kranz T., Baudner S. (1972). [Human serum proteins with high affinity to carboxymethylcellulose. II. Physico-chemical and immunological characterization of a histidine-rich 3,8S- 2 -glycoportein (CM-protein I)]. Hoppe. Seylers Z. Physiol. Chem..

[7] Katagiri M., Tsutsui K., Yamano T., Shimonishi Y., Ishibashi F. (1987). Interaction of heme with a synthetic peptide mimicking the putative heme-binding site of histidine-rich glycoprotein. Biochem. Biophys. Res. Commun..

[8] Leung L.L. (1986). Interaction of histidine-rich glycoprotein with fibrinogen and fibrin. J. Clin. Invest..

[9] Morgan W.T. (1981). Interactions of the histidine-rich glycoprotein of serum with metals. Biochemistry.

[10] Jones A.L., Poon I.K.H., Hulett M.D., Parish C.R. (2005). Histidine-rich glycoprotein specifically binds to necrotic cells via its amino-terminal domain and facilitates necrotic cell phagocytosis. J. Biol. Chem..

[11] Tsuchida-Straeten N., Ensslen S., Schäfer C., Wöltje M., Denecke B., Moser M. (2005). Enhanced blood coagulation and fibrinolysis in mice lacking histidine-rich glycoprotein (HRG). J. Thromb. Haemost..

[12] Olsson A.-K., Larsson H., Dixelius J., Johansson I., Lee C., Oellig C. (2004). A fragment of histidine-rich glycoprotein is a potent inhibitor of tumor vascularization. Cancer Res..

[13] Thulin A., Ringvall M., Dimberg A., Kårehed K., Väisänen T., Väisänen M.-R. (2009). Activated platelets provide a functional microenvironment for the antiangiogenic fragment of histidine-rich glycoprotein. Mol. Cancer Res..

[14] Kärrlander M., Lindberg N., Olofsson T., Kastemar M., Olsson A.-K., Uhrbom L. (2009). Histidine-rich glycoprotein can prevent Development of Mouse experimental Glioblastoma. PLoS One.

[15] Wakabayashi S., Koide T. (2011). Histidine-rich glycoprotein: a possible modulator of coagulation and fibrinolysis. Semin. Thromb. Hemost..

[16] Poon I.K.H., Olsson A.-K., Hulett M.D., Parish C.R. (2009). Regulation of histidine-rich glycoprotein (HRG) function via plasmin-mediated proteolytic cleavage. Biochem. J..

[17] Cunningham F., Allen J.E., Allen J., Alvarez-Jarreta J., Amode M.R., Armean I.M. (2022). Ensembl 2022. Nucleic Acids Res..

[18] Auton A., Brooks L.D., Durbin R.M., Garrison E.P., Kang H.M., Korbel J.O., 1000 Genomes Project Consortium (2015). A global reference for human genetic variation. Nature.

[19] Zou Y., van Breukelen B., Pronker M., Reiding K., Heck A.J.R. (2023). Proteogenomic features of the highly polymorphic histidine-rich glycoprotein Arose late in Evolution. Mol. Cell. Proteomics.

[20] Zhang Q., Jiang K., Li Y., Gao D., Sun L., Zhang S. (2015). Histidine-rich glycoprotein function in hepatocellular carcinoma depends on its N-glycosylation status, and it regulates cell proliferation by inhibiting Erk1/2 phosphorylation. Oncotarget.

[21] Jones A.L., Hulett M.D., Parish C.R. (2005). Histidine-rich glycoprotein: a novel adaptor protein in plasma that modulates the immune, vascular and coagulation systems. Immunol. Cell Biol..

[22] Liu Y., He J., Li C., Benitez R., Fu S., Marrero J. (2010). Identification and confirmation of biomarkers using an integrated platform for quantitative analysis of glycoproteins and their glycosylations. J. Proteome Res..

[23] Wu J., Xie X., Liu Y., He J., Benitez R., Buckanovich R.J. (2012). Identification and confirmation of differentially expressed fucosylated glycoproteins in the serum of ovarian cancer patients using a lectin array and LC-MS/MS. J. Proteome Res..

[24] Nordqvist S., Kårehed K., Stavreus-Evers A., Akerud H. (2011). Histidine-rich glycoprotein polymorphism and pregnancy outcome: a pilot study. Reprod. Biomed. Online.

[25] Hong M.-G., Dodig-Crnković T., Chen X., Drobin K., Lee W., Wang Y. (2020). Profiles of histidine-rich glycoprotein associate with age and risk of all-cause mortality. Life Sci. Alliance.

[26] Kassaar O., McMahon S.A., Thompson R., Botting C.H., Naismith J.H., Stewart A.J. (2014). Crystal structure of histidine-rich glycoprotein N2 domain reveals redox activity at an interdomain disulfide bridge: implications for angiogenic regulation. Blood.

[27] Reiding K.R., Lin Y.-H., van Alphen F.P.J., Meijer A.B., Heck A.J.R. (2021). Neutrophil azurophilic granule glycoproteins are distinctively decorated by atypical pauci- and phosphomannose glycans. Commun. Biol..

[28] Chen M., Assis D.M., Benet M., McClung C.M., Gordon E.A., Ghose S. (2023). Comparative site-specific N-glycoproteome analysis reveals aberrant N-glycosylation and gives insights into mannose-6-phosphate pathway in cancer. Commun. Biol..

[29] Chen Z., Yu Q., Yu Q., Johnson J., Shipman R., Zhong X. (2021). In-depth site-specific analysis of N-glycoproteome in human Cerebrospinal Fluid and glycosylation Landscape changes in Alzheimer’s disease. Mol. Cell. Proteomics.

[30] Reiding K.R., Bondt A., Franc V., Heck A.J.R. (2018). The benefits of hybrid fragmentation methods for glycoproteomics. TrAC Trends Anal. Chem..

[31] Riley N.M., Malaker S.A., Driessen M.D., Bertozzi C.R. (2020). Optimal dissociation methods differ for N- and O-glycopeptides. J. Proteome Res..

[32] Trastoy B., Naegeli A., Anso I., Sjögren J., Guerin M.E. (2020). Structural basis of mammalian mucin processing by the human gut O-glycopeptidase OgpA from Akkermansia muciniphila. Nat. Commun..

[33] Kuroda K., Ishii K., Mihara Y., Kawanoue N., Wake H., Mori S. (2021). Histidine-rich glycoprotein as a prognostic biomarker for sepsis. Sci. Rep..

[34] Matboli M., Eissa S., Said H. (2014). Evaluation of histidine-rich glycoprotein tissue RNA and serum protein as novel markers for breast cancer. Med. Oncol..

[35] Ding H.-G., Zhou H.-F., Diao M.-Y., Xu Y., Pan Q.-M., Shen X.-H. (2018). A novel biomarker of serum Histidine-Rich Glycoprotein (HRG) for diagnosing and predicting prognosis of ventilator-associated pneumonia (VAP): a pilot study. Eur. Rev. Med. Pharmacol. Sci..

[36] Ernst G., Dantas E., Sabatté J., Caro F., Salvado A., Grynblat P. (2015). Histidine-rich glycoprotein and idiopathic pulmonary fibrosis. Respir. Med..

[37] Völlmy F., van den Toorn H., Zenezini Chiozzi R., Zucchetti O., Papi A., Volta C.A. (2021). A serum proteome signature to predict mortality in severe COVID-19 patients. Life Sci. Alliance..

[38] Geyer P.E., Arend F.M., Doll S., Louiset M.-L., Virreira Winter S., Müller-Reif J.B. (2021). High-resolution serum proteome trajectories in COVID-19 reveal patient-specific seroconversion. EMBO Mol. Med..

[39] Costa A.R., Rodrigues M.E., Henriques M., Oliveira R., Azeredo J. (2014). Glycosylation: impact, control and improvement during therapeutic protein production. Crit. Rev. Biotechnol..

[40] Campbell C., Stanley P. (1984). A dominant mutation to ricin resistance in Chinese hamster ovary cells induces UDP-GlcNAc:glycopeptide beta-4-N-acetylglucosaminyltransferase III activity. J. Biol. Chem..

[41] Sallustio S., Stanley P. (1989). Novel genetic instability associated with a developmental regulated glycosyltransferase locus in Chinese hamster ovary cells. Somat. Cell Mol. Genet..

[42] Lin Y.-H., Franc V., Heck A.J.R. (2018). Similar albeit not the same: in-depth analysis of proteoforms of human serum, bovine serum, and recombinant human fetuin. J. Proteome Res..

[43] Chin C.L., Goh J.B., Srinivasan H., Liu K.I., Gowher A., Shanmugam R. (2019). A human expression system based on HEK293 for the stable production of recombinant erythropoietin. Sci. Rep..

[44] Kannicht C., Ramström M., Kohla G., Tiemeyer M., Casademunt E., Walter O. (2013). Characterisation of the post-translational modifications of a novel, human cell line-derived recombinant human factor VIII. Thromb. Res..

[45] Magalhães A., Duarte H.O., Reis C.A. (2021). The role of O-glycosylation in human disease. Mol. Aspects Med..

[46] Goth C.K., Vakhrushev S.Y., Joshi H.J., Clausen H., Schjoldager K.T. (2018). Fine-tuning limited proteolysis: a major role for regulated site-specific O-glycosylation. Trends Biochem. Sci..

[47] Wandall H.H., Nielsen M.A.I., King-Smith S., de Haan N., Bagdonaite I. (2021). Global functions of O-glycosylation: promises and challenges in O-glycobiology. FEBS J..

[48] Varki A., Cummings R.D., Aebi M., Packer N.H., Seeberger P.H., Esko J.D. (2015). Symbol nomenclature for Graphical Representations of glycans. Glycobiology.

[49] Ceroni A., Maass K., Geyer H., Geyer R., Dell A., Haslam S.M. (2008). GlycoWorkbench: a tool for the computer-assisted annotation of mass spectra of glycans. J. Proteome Res..

[50] Perez-Riverol Y., Bai J., Bandla C., García-Seisdedos D., Hewapathirana S., Kamatchinathan S. (2022). The PRIDE database resources in 2022: a hub for mass spectrometry-based proteomics evidences. Nucleic Acids Res..

