# Supplementary material for: Genotype-dependent N-glycosylation and newly exposed O-glycosylation affect plasmin-induced cleavage of histidine-rich glycoprotein (HRG)
Source: J Biol Chem. 2024 Jan 24;300(3):105683. doi: 10.1016/j.jbc.2024.105683 (PMC10882129; doi:10.1016/j.jbc.2024.105683)
Supplement: Supporting Information [file mmc9.docx]

**Supplementary Material to**

Genotype-dependent *N*-glycosylation and newly exposed *O*-glycosylation affect plasmin-induced cleavage of histidine-rich glycoprotein (HRG)

Yang Zou1,2, Matti F. Pronker,1,2, J. Mirjam A. Damen1,2,

Albert J. R. Heck1,2 and Karli R. Reiding1,2

**1Biomolecular Mass Spectrometry and Proteomics**, Bijvoet Center for Biomolecular Research and Utrecht Institute for Pharmaceutical Sciences, Utrecht University, Padualaan 8, 3584 CH, Utrecht, The Netherlands ​

**2Netherlands Proteomics Center**, Padualaan 8, 3584 CH, Utrecht, The Netherlands

**Supplementary Data Excel files**

**Supplementary Data Excel file 1. HRG sequences describing the five dominant mutations with MAFs of more than 10%.**

**Supplementary Data Excel file 2. Curation of the Byonic PSMs of HRG *N*-glycopeptides achieved by HCD and sHCD fragmentations with a score threshold of 150 and |Log Prob| threshold of 2, as well as controlling the FDR to be less than 0.01.**

**Supplementary Data Excel file 3. Curation of the Byonic PSMs of HRG *O*-glycopeptides achieved by HCD, sHCD, and EthcD fragmentations with a score threshold of 150 and |Log Prob|threshold of 2 and an FDR of less than 0.01.**

**Supplementary Data Excel file 4. Skyline MS1 peak areas of *O*-glycopeptides from HRG samples after OpeRATOR and SialEXO treatment.** The data of HEK293-rHRG-PI and HEK293-rHRG-SN are not shown since there was no data detected.

**Supplementary Data Excel file 5. Skyline MS1 peak areas of HRG *O*-glycopeptides.**

**Supplementary Data Excel file 6. Assignment of HRG fragments following plasmin-induced cleavage of HRG variants.** The mass calculation is based on the human plasma endogenous HRG. The areas and percentages of the bands from the gel as well as the t-test calculated results are also shown.

**Supplementary Data Excel file 7. Glycan compositions and glycan database used in the glycoproteomics searches.**

**Supplementary Data Excel file 8. Overview of the most abundant peptides with all the targeted glycosites.** The sequences from top to bottom are the most frequently detected peptides with all the targeted *N*-glycosites, and *O*-glycosites cleaved by trypsin, and the most frequently detected peptide sequences cleaved by different *O*-glycosites treated with trypsin, SialEXO and OpeRATOR.

**Supplementary Figures**


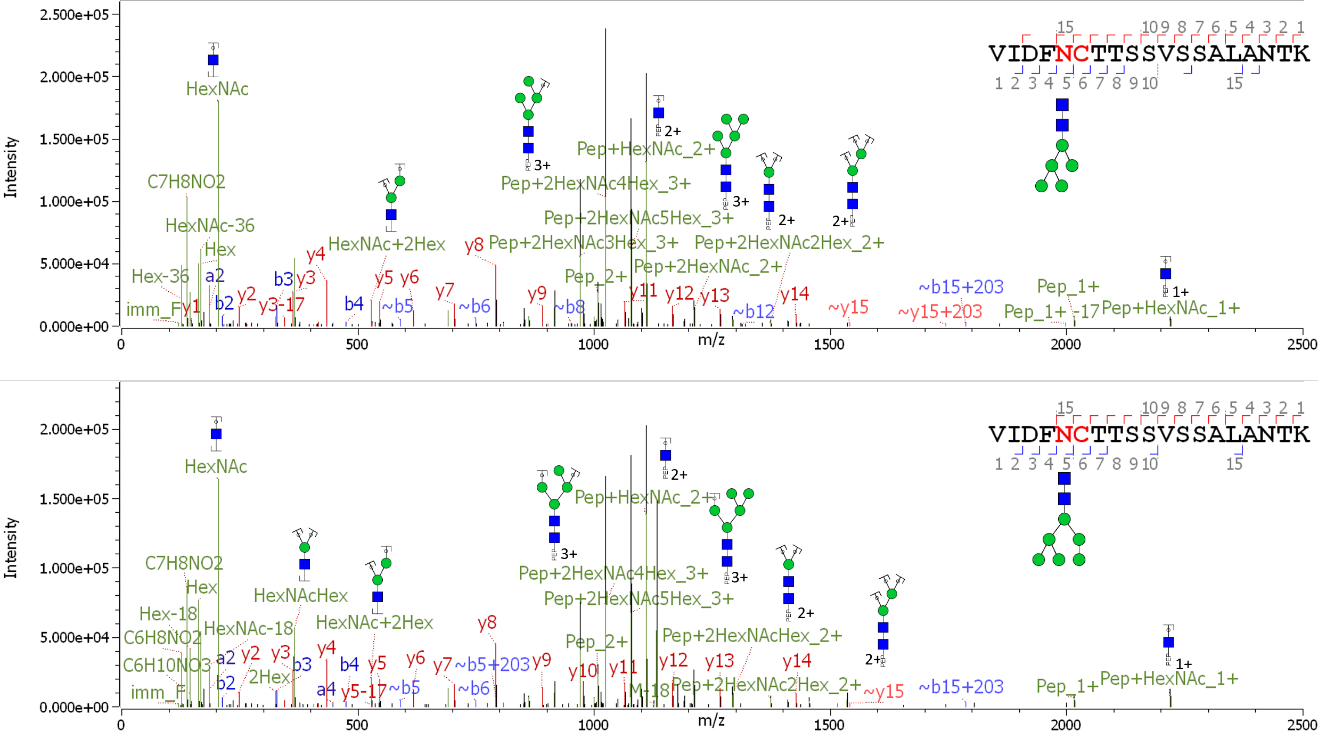


**Figure S1. Prototypical MS/MS spectra illustrative for HRG glycopeptides occupied with high mannose glycans.** The glycopeptides originate from recombinant CHO-cell derived HRG and cover Asn125. Proof of high mannose glycosylation is provided, among others, by the fragment ions at *m/z* 1023.7878 ([Pep + N2H4 + 3H]^3+^) and *m/z* 1077.8054 ([Pep + N2H5 + 3H]^3+^).


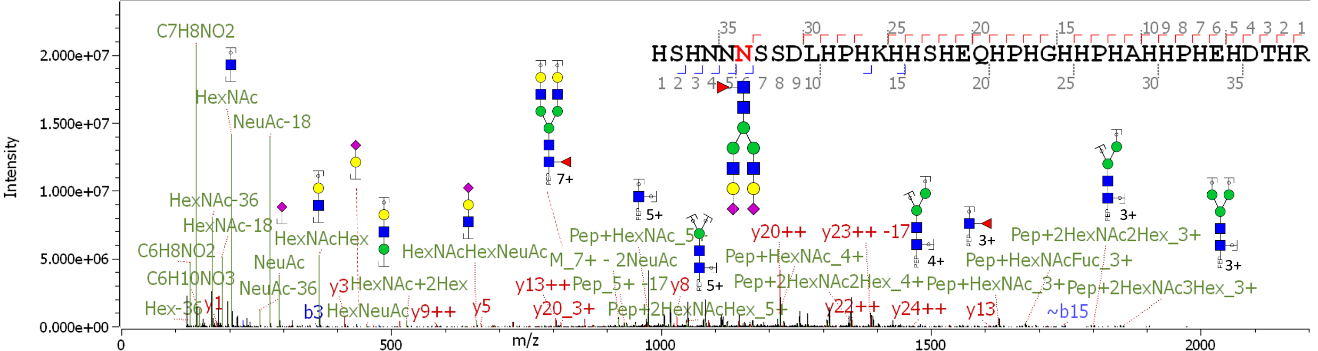


**Figure S2. Prototypical MS/MS spectrum illustrative for HRG glycopeptides occupied with core fucose glycans.** The glycopeptides originate from endogenous plasma HRG and cover Asn344. Proof of core fucose glycosylation is provided, among others, by the ions at *m/z* 920.6786 ([M - S2 + 7H]^7+^) and *m/z* 1673.7397 ([Pep + H1F1 + 3H]^3+^).


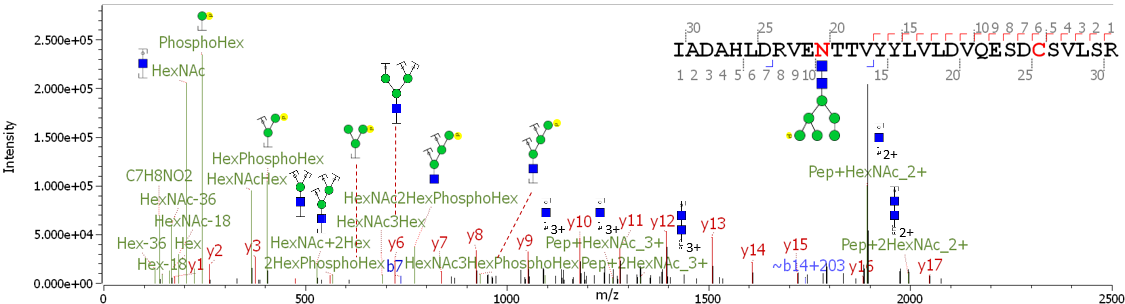


**Figure S3. Prototypical MS/MS spectrum illustrative for HRG glycopeptides occupied with phosphomannose glycans.** The glycopeptides originated from recombinant HRG from HEK293 cells (Pro204/Asn493) and covered Asn63. Proof of phosphomannose glycosylation is provided, among others, by the ions at *m/z* 243.0264 ([H1P1 + H]^+^), *m/z* 405.0793 ([H2P1 + H]^+^), *m/z* 567.1321 ([H3P1 + H]^+^), *m/z* 770.2114 ([N1H3P1 + H]^+^) and *m/z* 932.2643 ([N1H4P1 + H]^+^).


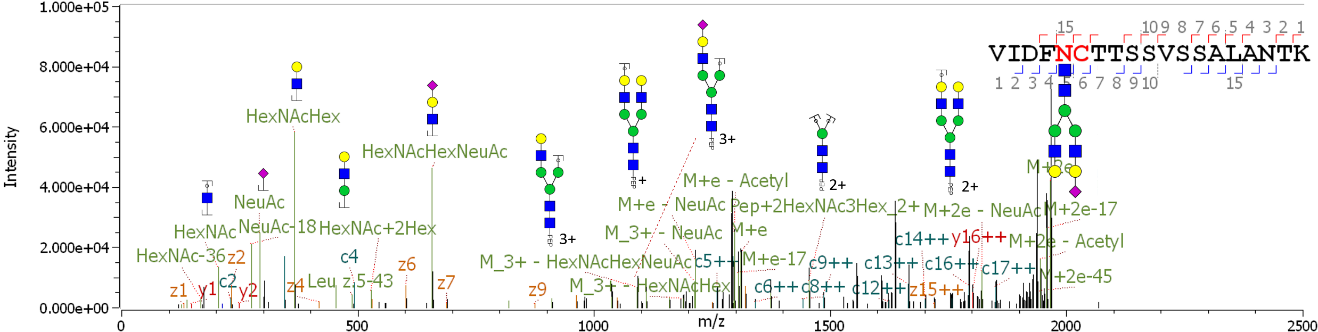


**Figure S4. Prototypical MS/MS spectrum illustrative for HRG glycopeptides occupied with complex diantennary glycans.** The glycopeptides originate from endogenous plasma HRG and cover Asn63. Proof of complex diantennary glycosylation is provided, among others, by the ions at *m/z* 675.2349 (N1H1S1), *m/z* 1091.4809 ([M - N1H1S1 + 3H]^3+^), and *m/z* 1213.1916 ([M-S1 + 3H]^3+^).


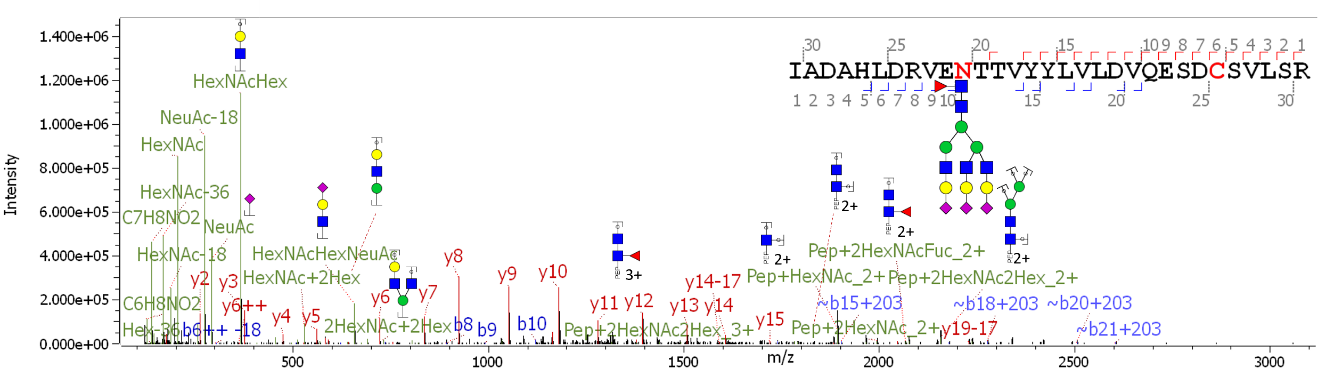


**Figure S5. Prototypical MS/MS spectrum illustrative for HRG glycopeptides occupied with complex triantennary glycans.** The glycopeptides originate from endogenous plasma HRG and cover Asn63. Proof of complex triantennary glycosylation is provided, among others, by the fragment ions at *m/z* 675.2349 (N1H1S1), *m/z* 1378.3300 ([Pep + N2F1 + 3H]^3+^) and *m/z* 2066.9913 ([Pep + N2F1 + 2H]^2+^).


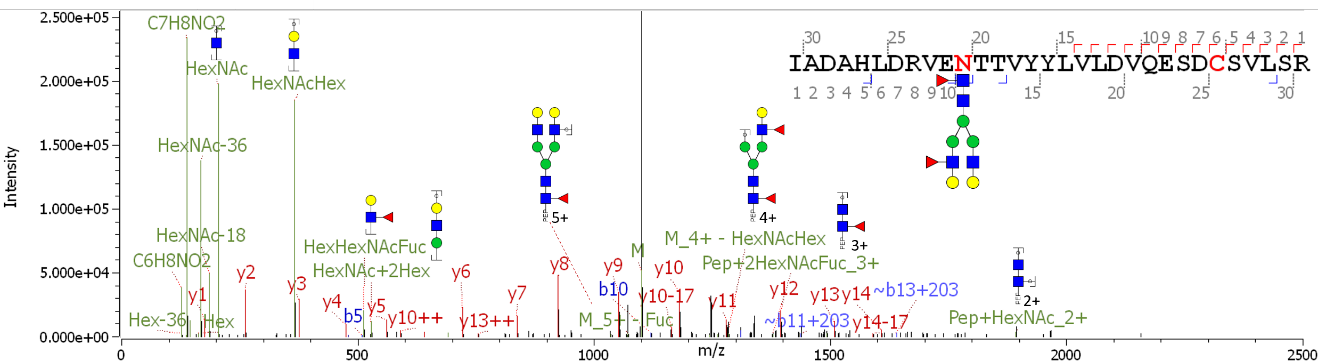


**Figure S6. Prototypical MS/MS spectrum illustrative for HRG glycopeptides occupied with antennary fucose glycans.** The glycopeptides originate from recombinant HRG from HEK293 cells (Ser204/Ile493) and cover Asn63. Proof of antennary fucose glycosylation is provided, among others, by the ions at *m/z* 512.1974 (N1N1F1), *m/z* 1070.6855 ([M - F1 +_5H]^5+^) and *m/z* 1283.3365 ([M – N1H1 + 4H]^4+^).


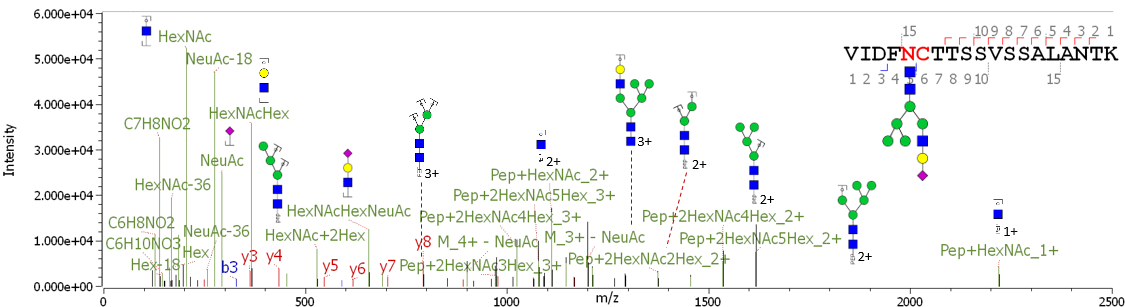


**Figure S7. Prototypical MS/MS spectrum illustrative for HRG glycopeptides occupied with hybrid glycans.** The glycopeptides originated from recombinant HRG from CHO cells and cover Asn125. Proof of hybrid glycosylationis provided, among others, by the ions at *m/z* 675.2349 (N1H1S1), *m/z* 1199.5161 ([M – S1 + 3H]^3+^), *m/z* 1535.1780 ([Pep + N2H4 + 2H]^2+^) and *m/z* 1616.2044 ([Pep + N2H5 + 2H]^2+^).


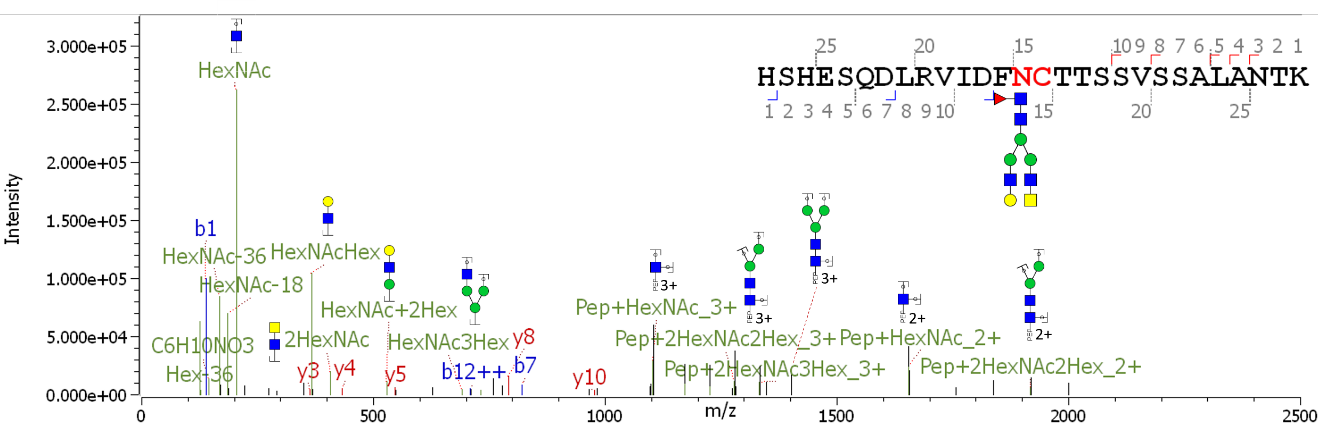


**Figure S8. Prototypical MS/MS spectrum illustrative for HRG glycopeptides occupied with LacDiNAc glycans.** The glycopeptides originate from recombinant HRG from HEK293 cells (Pro204/Asn493) and cover Asn63. Proof of LacDiNAc glycosylation is provided, among others, by the ions at *m/z* 407.1660 ([N2 + H]^+^).


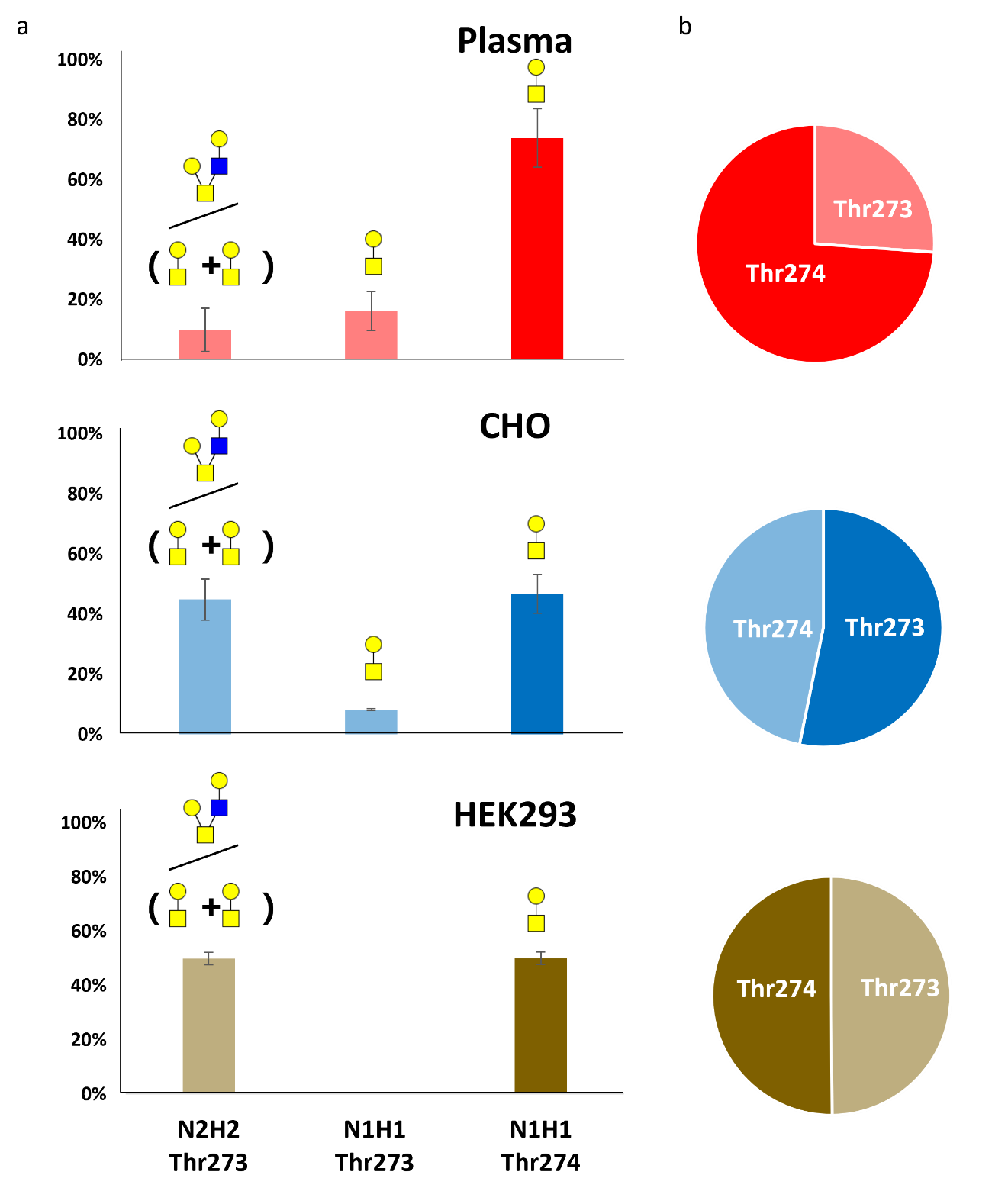


**Figure S9. Site localization distribution of the two new *O*-glycosylation sites.** From top to bottom are data originating from plasma HRG, CHO cells and HEK293 cells derived HRG respectively. **a)** The dominant *O*-glycopeptide distribution observed among these HRG variants. **b)** Site localization distribution among these HRG variants.


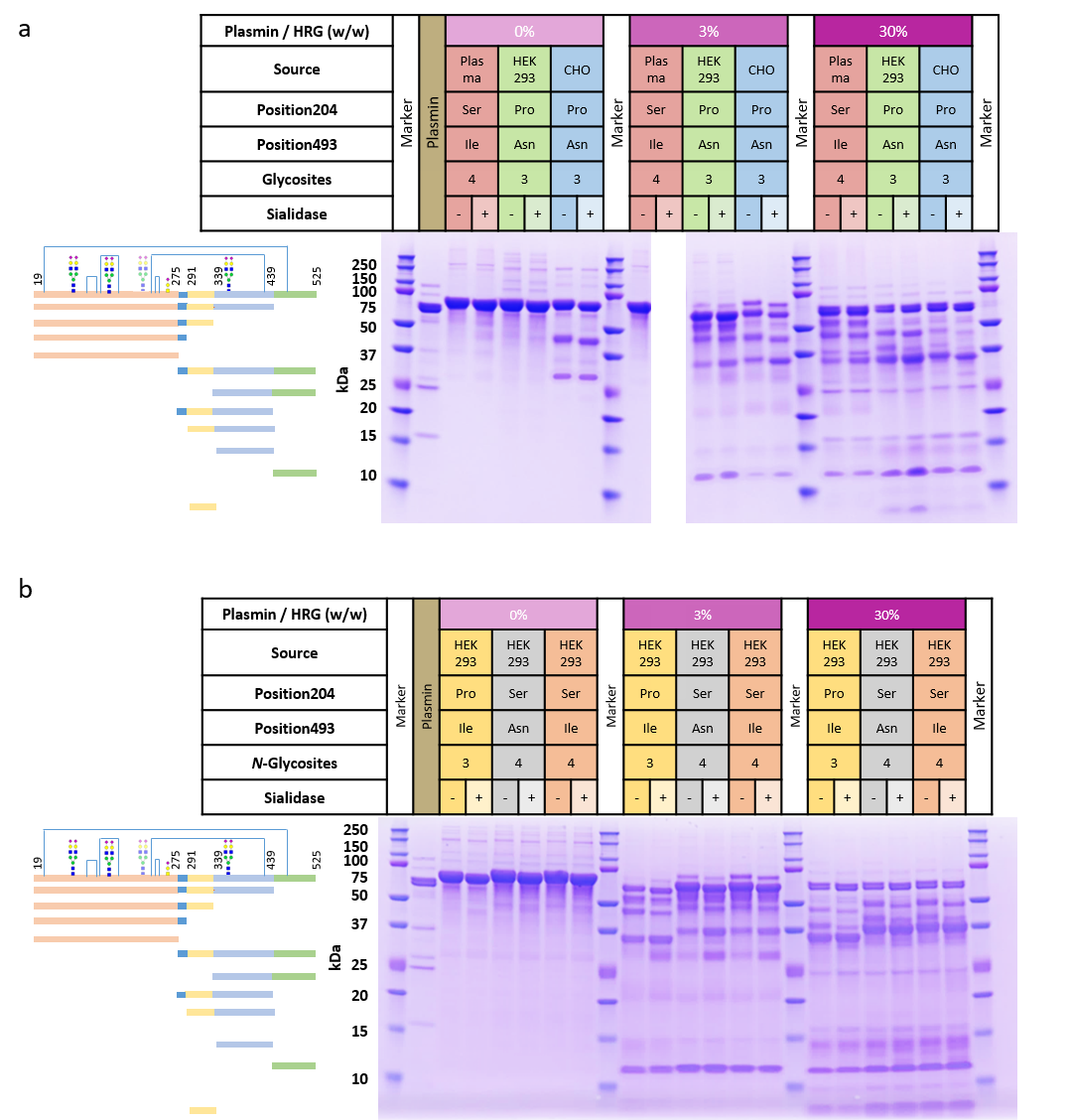


**Figure S10. Reducing gels of HRG samples incubated with plasmin (0%, 3% and 30%) for 1 h. a)** Gels for HRG originating from either plasma, HEK293 cells (Pro204/Asn493) or CHO cells. The gel lane of the plasma sample with the treatment of SialEXO and 3% plasmin was missing from the analysis but can be found in in **Figure 5** instead. **b)** Gel for recombinant HRG from HEK293 cell lines having distinctive mutations (Pro204/Ile493, Ser204/Asn493, Ser204/ Ile493). The fragment assignment is provided in **Supplementary Data Excel file 6.** The schematic in the left panel of the gels depicts the HRG fragment assignment after plasmin treatment. Fragments of different colors represent the segments that can potentially be generated by plasmin-cleavage at Lys275, Lys291, Arg339 and Arg439. The displayed glycosylations are the most abundant glycan observed on each glycosite from the plasma endogenous HRG, the blue lines depict disulfide bridges.
